# Water and Ion Channels: Crucial in the Initiation and Progression of Apoptosis in Central Nervous System?

**DOI:** 10.2174/157015908784533879

**Published:** 2008-06

**Authors:** Minghui Jessica Chen, Sugunavathi Sepramaniam, Arunmozhiarasi Armugam, Meng Shyan Choy, Jayapal Manikandan, Alirio J Melendez, Kandiah Jeyaseelan, Nam Sang Cheung

**Affiliations:** 1 Departments of Biochemistry, Yong Loo Lin School of Medicine, National University of Singapore, Singapore 117597, Singapore; 2 Physiology, Yong Loo Lin School of Medicine, National University of Singapore, Singapore 117597, Singapore

**Keywords:** Aquaporins, ion channels, apoptosis, apoptotic volume decrease.

## Abstract

Programmed cell death (PCD), is a highly regulated and sophisticated cellular mechanism that commits cell to isolated death fate. PCD has been implicated in the pathogenesis of numerous neurodegenerative disorders. Countless molecular events underlie this phenomenon, with each playing a crucial role in death commitment. A precedent event, apoptotic volume decrease (AVD), is ubiquitously observed in various forms of PCD induced by different cellular insults. Under physiological conditions, cells when subjected to osmotic fluctuations will undergo regulatory volume increase/decrease (RVI/RVD) to achieve homeostatic balance with neurons in the brain being additionally protected by the blood-brain-barrier. However, during AVD following apoptotic trigger, cell undergoes anistonic shrinkage that involves the loss of water and ions, particularly monovalent ions e.g. K^+^, Na^+^ and Cl^-^. It is worthwhile to concentrate on the molecular implications underlying the loss of these cellular components which posed to be significant and crucial in the successful propagation of the apoptotic signals. Microarray and real-time PCR analyses demonstrated several ion and water channel genes are regulated upon the onset of lactacystin (a proteosomal inhibitor)-mediated apoptosis. A time course study revealed that gene expressions of water and ion channels are being modulated just prior to apoptosis, some of which are aquaporin 4 and 9, potassium channels and chloride channels. In this review, we shall looked into the molecular protein machineries involved in the execution of AVD in the central nervous system (CNS), and focus on the significance of movements of each cellular component in affecting PCD commitment, thus provide some pharmacological advantages in the global apoptotic cell death.

## INTRODUCTION

The mechanism of cell death is classified into necrosis and apoptosis. Necrosis is commonly caused due to injury or cell damage or exposure to toxic substances. Cells undergoing necrosis swell, the cellular contents leak out causing damage to the surrounding cells and resulting in inflammation. In contrast cells undergoing apoptosis shrink, the DNA gets broken down, mitochondria releases cytochrome C, cells exhibit “eat me” signals such as phosphatidylserine and they break up into small membrane wrapped fragments [157]. This orderly process is often termed as programmed cell death (PCD). PCD, also known as apoptosis, usually occurs when there is a need to destroy cells that represent a threat to the organism itself without triggering massive inflammation. This could be virus-infected cells, DNA damaged cells, cancerous cells or even effector cells of the immune system. PCD is also necessary for proper development such as the sloughing off of the endometrium at the start of menstruation or for the formation of proper synapses between neurons in the brain. As such, PCD is an efficient and coordinated physiological mode of cell death adopted by the body to remove unwanted or dying cells at certain stage of body growth or in response to intrinsic or extrinsic stimuli in the absence of inflammation. It is characterized and identified by its unique and exclusive morphological features such as cell shrinkage, membrane blebbing, nuclear condensation, internucleosomal DNA fragmentation, externalization of phosphatidylserine and formation of apoptotic bodies, and biochemical states, such as activation of caspases and endonucleases, mitochondrial membrane depolarization and loss of cytochrome c.

However an imbalance of this orderly process can often result in various disease states. In fact, apoptosis is a common mode of cell death frequently observed in various neurodegenerative diseases. Increased apoptosis within the central nervous system is associated with neurodegenerative diseases such as Alzheimer’s disease (AD) and Parkinson’s disease (PD) in which a massive progressive loss of different populations of neurons is observed [155], whereas a decreased apoptotic event within the haemopoietic system is implicated with leukaemia and lymphoma [110]. 

Triggers of PCD are usually the absence of survival factors or the presence of negative signals. PCD occurs *via* three known modes. It can be triggered *via* an intrinsic or extrinsic stimulus or *via* reactive oxidative species (ROS). Intrinsic stimulus includes proteins such as Bcl-2, Bax and cytochrome C. This usually results in apoptosis *via* the mitochondrial pathway whereas extrinsic stimuli are molecules such as the Fas Ligand (FasL) and Tumor Necrosis Factor (TNF) which bind to their respective receptors and initiate apoptosis *via* the death receptor pathway. ROS such as hydroxyl radicals or superoxide can cause direct damage to the lipid membranes and nucleic acids. 

Though occurrence of PCD could be ascertained together by various apoptotic characteristics, cell shrinkage as a pre-cedent event of apoptosis is a single exclusive feature consistently observed in all physiologic apoptotic models. In the recent years, a new term ‘apoptotic volume decrease’ (AVD) has been coined to denote the loss of cellular volume in the absence of osmotic imbalance in PCD resulting in visible morphological cell shrinkage observation. Cell shrinkage is a ubiquitous and fundamental feature of programmed cell death that is independent of the apoptotic insults and conserved among species. Various drug-induced apoptotic models to mimic the pathological conditions of neurodegenerative disorders such as AD and PD ubiquitously demonstrated the occurrence of cell shrinkage. In a healthy brain, the presence of glial cells and blood-brain-barrier allows buffering of any osmotic fluctuations in the brain during normal physiological neuronal activity. 

Under normal physiological conditions, animal cells are able to withstand sudden osmotic changes in the surrounding due to the presence of regulatory mechanisms. Animal cells have incorporated mechanisms including ion channels, plasma membrane transporters and cytoskeletal reorganization to compensate for cell volume changes. Thus they are able to sense and respond to changes in cellular osmolarity rapidly. Active innate processes, known as regulatory volume increase (RVI) and regulatory volume decrease (RVD), are consistently in check to regulate any cell shrinkage or swelling respectively under anisotonic conditions, so as to restore cells to their resting volumes for maintenance of normal cellular metabolism and survival [71]. This involves short and long-term changes to ion concentrations, especially K^+^, Na^+^, Cl^–^, H^+^ and HCO_3_^-^, transport of osmolytes amino acids, methylamines, polyols, sugars and urea and also changes in gene expression. [100, 105]. Regulation during RVI is maintained by the inward movement of solutes predominately K^+^ whereas regulation during RVD is attained mainly by the outward movement of Cl^-^ ions. The movement of water across the membrane is largely controlled by these intracellular ions and osmotically active substrates. For instance, during hypertonic stress, cells rapidly shrink. However, RVI mechanisms permit cells to recover their steady-state volume [65, 77]. The Na^+^/H^+^ antiporter and teams up with the Na^+^/K^+^/2Cl^– ^co-transporter and Cl^–^/HCO_3_^-^ exchanger producing a net influx of NaCl and water [100]. The cell counters this increase in intracellular Na^+^, with the help of Na^+^/K^+^-ATPase where it exchanges Na^+^ for K^+^ at a much higher rate, and thus provides an electrogenic gradient by maintaining low intracellular Na^+^ and, secondarily, a low Cl^–^ concentration [37, 38, 100].

During patho-physiological conditions, neurons may inevitably be exposed to anisotonic situations that can severely disrupt neuronal activity and increase vulnerability to seizures and hypoxic spreading depression, as such triggering the RVI or RVD rescuing responses. In the event of pathological conditions when cells are committed to apoptosis, an AVD response is induced under isotonic condition, firing off a chain of reactions leading the cell to its irreversible death fate. Initially perceived to be a passive, secondary feature of apoptosis, AVD, now proved to be a critical precedent molecular event that involved dysregulation of ionic and water homeostasis and cytoskeletal reorganization and subsequent progression of apoptosis, e.g. caspase and nuclease activation. Significant effluxes of primary intracellular ions e.g. K^+^, Na^+^ and Cl^-^, together with loss of cellular water underlie the fundamental of AVD, with each having a role to play in creating a hypotonic environment within the cell and committing the cell to die. In this review, we will focus on the mechanistic events underlying AVD, particularly the significance of real-time movements of ions and water throughout the event of programmed cell death in the central nervous system (CNS).

## ROLE OF AQUAPORINS

Aquaporins (AQP) are a family of small hydrophobic, integral membrane proteins ranging from 26 to 34kDa in size. They are expressed in all living organisms with the overall primary sequences of AQPs showing about 19 to 52% homology. To date, 13 different AQPs have been identified in mammals, AQP 0-12. These integral proteins have been found to form transmembrane water channels that play critical roles in controlling the water flow into and out of cells [1]. Although initially thought to be facilitators of efficient water permeation, recent studies are indicating that several of the AQPs are also capable of permeating other small solutes such as anions, urea and glycerol and thus eventually leading to their classification. Thus although originally implicated in water transport, the roles of AQPs has expanded considerably. Based on their permeability characteristics AQPs are classified into two categories namely classical AQPs and aquaglyceroporins. AQP 0, 1, 2, 4, 5, 6, and 8 are considered to be permeable to water and thus grouped as classical AQPs [1]. Both AQP6 and AQP8 are also permeable to several other molecules. AQP6 has been proposed to be permeable to chloride and nitrate ions in a pH sensitive manner whereas AQP8 has been found to be permeable to urea [145, 146]. Although functional studies indicate almost all AQPs are capable of permeating water at varying degrees, AQPs 3, 7, 9 & 10 are considered as aquaglyceroporins. These channels are found to be mainly permeable to glycerol. They also allow the passage of other small solutes such as urea [1]. The two most recent additions to the AQP family, AQP11 and AQP12 are categorized as subgroups of AQPs due to the fact that they contain a variant amino acid sequence at the point where the polypeptide folds to form the water channel (see structure and water movement in AQPs). Just recently AQP11 with the variant NPA box has also been shown to be permeable to water indicating that the conserved NPA motif might not be crucial for water movement [142]. The permeability characteristic of AQP12 has not been determined yet. 

## STRUCTURE AND WATER MOVEMENT IN AQPS

The structure of an AQP molecule consists of six membrane spanning segments with three extracellular and two intracellular loops with the amino and carboxyl ends of the monomer being embedded within the cytoplasm [117]. The intracellular loops fold into the plasma membrane to form the water pores or hemipores. Within the hemipores exist the two highly conserved hydrophobic amino-acid sequences, Asparagine-Proline-Alanine (NPA), considered crucial for the movement of water molecules [64]. Each AQP monomer folds into a structure representing that of an hour-glass, to form an independent water channel by itself. Yet freeze-fracture studies show that AQPs exists as a homotetramer within the plasma membrane [130]. Thus each AQP contains four functional monomers which allow for independent water movement. Water movement across the AQP water channels have been postulated to be *via* size restriction and electrostatic repulsion [66]. The course through which the water molecules move through is narrow enough to physically restrict molecules larger than water itself. Furthermore the narrow pathway allows for the passage of only a single water molecule at any one time point. Simultaneously the hydrophilic pore, created by the presence of conserved and positively charged arginine and histidine residues, repel the entry of other positively charged molecules or protons into the water channel [2].

## AQPs IN CNS

So far seven AQPs; AQP1, AQP3, AQP4, AQP5, AQP8, AQP9 and AQP11 have been identified in various animal tissues in the CNS. *Via* real-time studies, including the above mentioned AQPs, we also observed the expression of AQP6 and AQP7 in mouse cortical neurons. 

Expression of AQP1 is restricted to the apical microvilli of the villous like processes of the epithelial cells of the rat choroids plexus [40]. It is not observed in the basolateral membranes of the epithelial cells or the endothelial cells of the underlying fenestrated capillaries [41, 93]. Choroid plexus is the site of cerebrospinal fluid production, the concentration of AQP1 in the apical membrane of its epithelial cells suggests that AQP1 may be involved in the secretion of cerebrospinal fluid. Reduced expression of AQP1 and its recovery after space flight was reported, suggesting the regulation of the AQP1 expression by gravity [80].

AQP4 is one of the water channels highly expressed in the brain. AQP4 mRNA was found to be ten times higher in the rat brain compared to the eye, kidney, intestine, or lung [64, 127]. Due to two different translation initiating methionines, two different isoforms of AQP4 have been shown to exist [64]. Both isoforms are found in the brain. AQP4 is highly expressed in the plasma membrane of astrocytes. It is plentiful in astrocyte cells bordering the subarachnoidal space, ventricles, and blood vessels. High levels of AQP4 were noted in areas where astrocytes come into direct contact with capillaries, ependymal layer and pia. In addition, the basolateral membrane of the ependymal cells that line the subfornical organ is positive for AQP4 [94]. The sites of AQP expression in the brain suggest a role in the movement of water across the blood brain barrier and thus in cerebrospinal fluid dynamics (CSF) and the formation of brain edema [13, 17, 64]. More recently, low but significant AQP4 mRNA expression has been associated to the choroid plexus, which is associated to CSF formation [129]. Immunoelectron microscopy analysis has failed to detect AQP4 protein within neurons [88, 94, 138]. However, employment of *in situ* hybridization has been positive for significant localization of AQP4 mRNA within neurons although the hybridization signal is low [129]. 

AQP9 is a water channel with broad substrate specificity; highly permeable to water and other non-charged small solutes such as glycerol, urea, lactate, arsentite purines and pyrimidines [53, 126]. The high permeability rate of AQP9 to water and small solutes has implicated its role in rapid cellular uptake and exit of metabolites with minimal osmotic perturbation. Astrocytes in the brain have been shown to express AQP9 whose expression levels are upregulated during ischemia [126] implicating a role in the regulation of postischemic edema in the brain [4]. Immunohistochemical staining also showed that AQP9 is present in tanycytes localized in the area lacking the blood–brain barrier such as the circumventricular organs of the third ventricle and hypothalamic regions, suggesting its possible role in the osmoreception [92]. AQP9 immunolabeling was found on astrocyte processes in the periventricular region of parenchyma and in the glia limitans, bordering the subarachnoid space [4]. AQP4 is also present in these regions, suggesting that AQP9 contributes with AQP4 to the facilitation of water movements between CSF and brain parenchyma. In addition, AQP4 and AQP9 labeling on astrocytes was found in white matter tracts, hippocampus, septum, and hypothalamic nuclei including magnocellular nuclei [4]. The existence of twoisoforms of AQP9 was reported in brain mitochondria suggesting a possible role in mitochondrial membrane permeability [18]. Yet, another study rebutted these findings [145]. 

AQP3, 5, and 8 expression was detected in whole rat brain and astrocytes *via* reverse transcription–polymerase chain reaction. The presence of AQP3 in the ependymal cells was also reported [76]. By immunofluorescence labeling, AQP3 was localized to the basolateral membrane. AQP8 expression was also found in neurons and oligodendrocytes whereas AQP5 expression was only found in neurons [143]. Transient upregulation of AQP5 in hypoxic brain suggested a possible role in inducing edema. AQP11 has a unique distribution in brain, appearing in Purkinje cell dendrites, hippocampal neurons of CA1 and CA2, and cerebral cortical neurons [39]. AQP11 has been found to be localized intracelluarly in the endoplasmic reticulum of rat brain [85]. Though initially thought to be a non-water permeable channel, a very recent report suggests otherwise [142]. The physiologic role of these proteins in the brain remains to be determined.

## AQPs AND APOPTOSIS

Ever since the incidental discovery of AQPs, they have been implicated in various diseases such as brain edema, cancer, nephrogenic diabetes insipidus and, and eventually associated with cellular apoptosis [59]. Although the role of AQPs in apoptosis has been implicated in AVD, the exact mechanisms that trigger the changes in the cellular volume remain to be elucidated. Here we look at the several studies that have been done to establish a link between apoptosis and AQPs. 

The direction of water flow in or out of the cell is determined by the intracellular ion concentrations and osmotically active substrates. During hypertonic stress, when the cells rapidly shrink, RVI mechanisms kick in [65, 77]. The increased expression and prolonged half-life of AQPs in cells exhibiting hypertonic stress seems to indicate that water movement during stress conditions are mediated *via* AQPs [73]. The half-life of AQP is regulated *via* ubiquitination (Ub). Ub-dependent pathways have been shown to play major roles in a various biological processes, some of which are, cell differentiation, DNA repair, transmembrane and vesicular transport, stress responses and apoptosis [43,44]. Once the target protein is ubiquitinated, it gets degraded by the protoesome. 

AQPs have also been shown to be ubiquitinated and eventually degraded by proteosomes. Under stressful conditions cells tend to reduce protein synthesis and increase ubiquitination. However [73] showed that under hypertonic stress, while total cellular ubiquitination increased, AQP1 exhibited decreased ubiquitination and thus a prolonged half-life of over 24 h. In fact the group showed that the reduction in ubiquitination occurred in parallel with an increase in total AQP1 protein expression. This observation seems to indicate the importance of functional AQP1 during hypertonic conditions. 

**Fig. (1):**
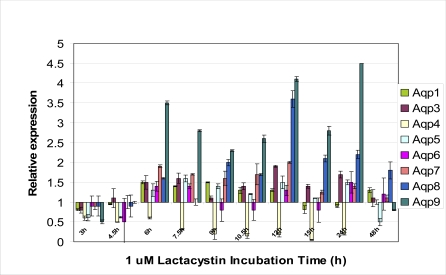
Real-time PCR analysis of the temporal change in gene expression of various aquaporins during 1 µM lactacystin treatment in cultured murine cortical neurons.

Proteasomal dysfunction is observed in the onset of cellular toxicity in numerous neurodegenerative diseases, and has been postulated as a probable mechanism leading to their pathogenesis. To date, numerous studies have demonstrated a negative correlation between proteasomal activity and severity of these disorders [22, 125]. As such, the involvement of signaling pathways downstream of proteasomal inhibition evokes curiosity. Classical proteasome inhibitors such as lactacystin, MG132 etc, have been commonly employed to induce neuronal toxicity, where cells undergo apoptosis [22, 120, 147]. Employing real-time PCR technique, our data demonstrated that cortical neurons induced to undergo apoptosis *via* lactacystin treatment showed increased AQP1 expression at 6 h (Fig. **1**). The increased expression was sustained up to 12 h after which it was downregulated. Onset of apoptosis in lactacystin-treated neurons occurred at 15 h. Although AQP4 is considered to be the main water channel in the brain, surprisingly it was highly downregulated throughout lactacystin induced apoptosis suggesting that loss of water during AVD might occur *via* other AQPs. The downregulation of AQP4 gene was also observed in microarray studies (Table **1**). Microarray analysis was conducted using Affymetrix Murine U74A Genechips (n=3 for each time-point) to investigate temporal expression profiles of lactacystin-treated primary murine cortical neurons containing probes for 12 488 genes and expressed sequence tags. Gene profiling analysis followed up to 48 h using 1µM lactacystin treatment was selected in an attempt to identify genes with significant differential gene expression levels during cell commitment to apoptosis. Differential gene regulation using different doses of lactacystin was demonstrated in our previous microarray data [147]. All data were generated using cultures of mouse embryonic day 15-16 cortical neurons prepared as previously described [21,22].

While the increase in the half-life of the AQP1 protein might be in direct relation to the amount of water loss from the cell, it is also possible that it is a compensatory mechanism by the cell to allow for increased water uptake *via* the bidirectional channel to rectify the osmotic stress. Although whether the increased AQP1 expression observed during hypertonic stress is an effect of direct water loss or a compensatory mechanism is not clear, the importance of AQP in cellular volume exchange is evident. Thus it is no surprise that they play a crucial role in AVD. In fact it has been shown that the plasma membrane AQP activity can indeed affect the rate of apoptosis. Sterically blocking AQP1 activity with HgCl_2_ was shown to prevent AVD and the subsequent downstream apoptotic events such as cell shrinkage, DNA degradation, loss of mitochondrial membrane permeability and caspase 3 activation [54]. This trend was observable in various cell types indicating that the ability to block apoptosis through the inhibition of AQPs is not cell specific. The importance of AVD *via* AQP and subsequent apoptosis is further shown in a study by [56]. They showed that hepatic tumor cells exhibited decreased expression for AQP8 and 9 and showed a lack in water movement across the cellular membrane when compared to normal cells. These cancerous cells also exhibited inherent resistence to apoptotic stimuli. In fact lactacystin induced apoptotic mouse cortical neurons showed extremely high expression levels for AQP8 and AQP9 (Fig. **1**). These data suggests the need of a functional AQP channel during AVD. 

Although extrusion of cellular water during apoptosis is similar to cells undergoing hypertonic stress, the distinct feature is that apoptotic cells are unable to activate their RVI mechanism. While the reason for this remains to be elucidated, there has been some suggestions that perhaps the AQPs lining the plasma membrane are inactivated, thus shutting down the pathway for water influx and subsequent recovery. In fact [54] showed that the loss in K^+^ ions drives AVD *via* AQPs, after which the AQPs are inactivated. They believe that this inactivation process is crucial as it is necessary for the further decrease in K^+^ ion concentrations which will eventually result in the activation of apoptotic enzymes. Thus AQP inactivation after AVD is just as critical as AQP activation before AVD.

The mechanism of AQP inactivation is still unclear. However a recent study has suggested that in apoptotic cells, AQP1, 8 and 9 associate with caveolin-1 and are in due course inactivated [55]. Caveolin proteins function in the formation of caveolae which are flask-shaped invaginations of the plasma membrane. These proteins are though to sequester molecules and hold them in a dormant state. The authors showed that following AVD, AQPs are not degraded or removed from the plasma membrane. The other possibility of AQP inactivation could be *via* the cytoskeleton associated anchor protein fodrin. Fodrin functions as an anchor protein, holding a variety of other proteins including ion channels and transporters to the plasma membrane [91]. However, during apoptosis, this protein is cleaved in a caspase dependent manner, resulting in the loss of its function [79]. Thus there exists a possibility that like ion channels, AQPs are also anchored to the plasma membrane *via* fodrin and cleavage of fodrin during apoptosis renders the AQP non-functional. 

The role of mitochondria in apoptosis has also been extensively studied. Mitochondria are the bioenergetic and metabolic centers of eukaryotic cells. They seem to play the crucial role of balancing the life and death of a cell. Expansion and contraction of the mitochondrialmatrix are thenet result of the water movement which is linked to the movement of solutes, including K^+^ and Ca^2+^ ions, acrossthe inner mitochondrialmembrane (IMM) into and out of the mitochondria [35]. Althoughthe osmotic movement of water into and out of the mitochondrionis central for its morphology and activity, the molecular mechanismsand the pathways for water transport across the IMM, the main barrier for molecules moving into andout of the organelle, are completely unknown. 

A recent discovery of AQP expression in mitochondria has stirred considerable interest. The expression of AQP8 and 9 has been identified in mitochondria. An isoform of AQP9 has been found to in the brain mitochondria and thought to be involved in lactate transport by allowing the mitochondria to adjust to the metabolic state of the cytoplasm. The mitochondrialAQP9 is proposed to be a hallmark of astrocytes and midbrain dopaminergic neurons [3].AQP8 has been identified in the IMM of rat liver and murine central nervous system (CNS) cells and postulated to be involved in the maintaining the mitochondrial matrix volume which is crucial for the proper mitochondrial function [18]. 

**Fig. (2):**
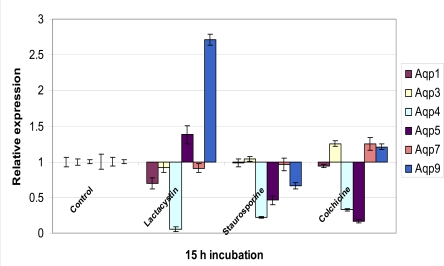
A real-time PCR analysis of the differential gene expression of the various aquaporins in cultured murine cortical neurons at 15 h treatment with 1 µM lactacystin, staurosporine and colchicines respectively.

Immunoblotting, electronmicroscopy, and biophysical studies showed that the highest expression for AQP8 was found in the largest mitochondria. However AQP inhibition studies showed only partial inhibition of osmotic water movement and thus indicated the presence of alternative water pathways. Hence the authors postulated that perhaps water movement *via* AQP8 might be important during times of rapid expansion of mitochondrial volume suchas those occurring during active oxidative phosphorylation andthose following apoptotic signals. They suggested that mitochondrial AQP8 might act independently or in association with thepostulated "permeability transition pore complex" that allows for the rapid entry of solutes and water into the matrix [128, 160]. This results in the expansion of the organelle, followed by the rupture of the outer mitochondrialmembrane, and release pro-apoptoticelements into the cytoplasm leading to apoptosis. This hypothesis was supported by the observation that in the presence of AQP blockers, treatment with apoptotic agent Cd^2+^ ion prevented the swelling of mitoplasts [72]. However, [145] recently contradicted these findings by showing that there was no siginificant AQP expression in the mitochondria. We observed AQP 8 and 9 gene expression in cortical neurons (Fig. **1**). In fact these two genes were highly upregulated after 6 h of lactacystin treatment and exhibited significantly high gene expression levels for 24 h. However this highly increased AQP9 expression was only observed in lactacystin induced apoptosis. Stauros-porine (a broad spectrum kinase inhibitor)-mediated apoptosis showed downregulation of all AQP genes whereas although colchicines (inhibitor of microtubule polymerization)-mediated apoptosis showed a similar AQP gene profile when compared to that of lactacystin-mediated apoptosis, the increase in AQP9 expression was marginal (Fig. **2**).

## ROLE OF POTASSIUM IONS

Cell body shrinkage, Bcl-2 family protein alteration, Bax activation, caspase-3 cleavage and endonuclease activation are typical apoptoticnmolecular events widely believed to be mediated by a dramatic imbalance of ion homeostasis, primarily due to loss of intracellular K^+^ *via* K^+^-permeable channels and/or dysfunction of the Na^+^/ K^+^-ATPase since K^+^ is the most dominant and osmotically crucial cation in the cytosol [84, 141, 149, 153]. Under normal physiological conditions, K^+^ acts as an endogenous modulator of the apoptotic checkpoints particularly as potential repressors of executioner caspases and nucleases involved in apoptosis [11, 46, 47]. Besides being an apoptotic repressor, maintenance of normal cellular K^+^ level is required to sustain cellular ion homeostasis required for normal cell volume maintenance *via* RVD or RVI [69, 150]. This is physiologically important as massive and uncompensated loss of intracellular K^+^ leads to AVD [78]. It has been demonstrated that apoptosis of mouse neocortical neurons was coupled to an early enhancement of delayed rectifier (IK) current with a loss of total intracellular K^+^, a phenomenon exclusive to apoptosis and not seen in neurons undergoing excitotoxic necrosis. As such, it is fairly appropriate to speculate K^+^ efflux is crucial in the mediation of neuronal apoptosis [153]. Various papers suggested a correlation between the exhibition of apoptotic characteristics with shrunken cell population, and that DNA degradation was almost exclusively observed in these cells [9, 11, 46, 96]. Interestingly, this DNA degradation could be inhibited by impeding the efflux of K^+^ through the application of high extracellular K^+^ concentration. Furthermore, [124] indicated that low intracellular K^+^ facilitated and enhanced the binding of NF-κB to target gene DNA thus upregulating its transcriptional activity without triggering its activation or translocation. It is demonstrated *in vitro* that low endogenous K^+^ promotes binding of pro-apoptotic transcription factors p53 and Forkhead and hinders the transcription activity of anti-apoptotic factors e.g. CREB protein [144]. A recent literature using cultured cerebrocortical neurons to study nitric oxide-mediated apoptosis demonstrated that only loss of K^+^ from the cell body was associated with cell shrinkage and membrane blebbing though K^+^ efflux was also observed to occur in the distal neuritis [12].

## ROLE OF PLASMA MEMBRANE K^+^ CHANNELS AND K^+^-PERMEABLE RECEPTORS

Under physiological state, K+ channels play an important role in the maintenance of excitability and relay of information within the central nervous system. Plasma membrane K+ channels have been well characterized at the molecular level to play important roles in synaptic plasticity and in the pathogenesis of numerous neurological disorders [57, 107]. Plasma membrane K+ channels in excitable cells can be categorized into five different classes: voltage-gated K+ (Kv) channels, Ca2+-activated K+ (KCa) channels, ATP-sensitive K+ (KATP) channels, inwardly rectifying K+ (Kir) channels, and two-pore domain K+ (K2P) channels, whose structural and biochemical properties are extensively reviewed in [16]. In this review, we shall focus on the roles of Kv, Kir and K2P channels in the central nervous system.

## KV CHANNELS

Existence of a large family of Kv channel genes has been found to be expressed in the mammalian brains. Kv channels are characterized by their effective inhibition by 4-amino-pyridine (4-AP), tetraethylammonium chloride (TEA), dendrotoxin and tityustoxin as well as by their sensitivity to membrane potential (Em) [16]. Kv channels are involved in the mediation of K^+^ efflux upon membrane depolari-zation down the electrochemical gradient. It is shown in rat fetal neurons, sulfhydryl-oxidizing agent 2,2-dithiodipyridine (DTDP) is able to induce apoptosis by triggering similar kinetic profiles of TEA- and 4-AP-sensitive K+ currents [84, 153]. In neuron-glial cultures, DTDP was shown to induce a delayed rectifier outward potassium currents through Kv2.1 [158]. In the mammalian CNS, it is known that Kv2.1 is expressed ubiquitously and at high levels throughout and is strongly localized on the soma and dendrites of both principal neurons and interneurons in the hippocampus [87]. Properties of Kv 2.1 such as oxygen sensitivity, inactivation kinetics, sensitivity to TEA, voltage-dependence are regulated by the electrically silent pore-forming (α or γ) subunits, such as Kv6.1, Kv8.1, and Kv9.3, a group which themselves do not form functional homotetrameric channels (i.e. inability to induce electrical voltage) but are involved in modulating activity of other functional α subunits e.g. by forming heterotetramer with members of Kv2 family to create current properties distinctively different from that of wild-type Kv2 channels [16]. Majority of the delayed rectifier potassium currents in neuronal cells are mainly mediated by the α-subunit of Kv2.1 [31]. Kv2.1 channel, member of a family of Kv channels is functionally involved in the repolarization of the membrane potential of excitable tissue during electrical activity, thus modulating cell firing. As such, Kv2.1 possesses a critical and dynamic role in the control of transmission of electrical signals into and out of the neuronal soma [86]. The increase in K^+^ outflow is protein synthesis independent and precedes caspase activation [84]. Instead the current surge is associated with the de novo insertion of additional functional Kv2.1 into the plasma membrane, following apoptotic stimulation. Paradoxically, it is reported that transcriptional upregulation of Kv2.1 has been associated with the enhancement of delayed rectifier outward potassium currents induced by beta-amyloid peptide 25-35 [60]. [101] further commented that Kv2.1-encoded K^+^ channels are critical for the activation of apoptotic signaling cascade in mammalian cortical neurons in culture treated with staurosporine and oxidant and are implicated in the increase in susceptibility to apoptogens in a non-excitable cell. A sudden surge in activity of Kv2.1 channels leads to massive outflow of cytoplasmic K^+^ ions and eventually apoptosis. Neurons deficient in functional Kv2.1-encoded K^+^ channels are protected against oxidant and staurosporine-mediated apoptosis [101]. Neuroprotection can also be achieved by application of a novel Kv2.1 channel blocker at non-toxic levels [158]. Nevertheless, activity of other subunits, such as Kv2.2 and Kv3.1/3.2, also accounts for generation of the delayed rectifier outward potassium currents in vertebrate neurons [116]. An increased expression of Kv1.2 channels was indicated in SH-SY5Y neuroblastoma cell line following hypoxia and glucose deprivation, with similar trend observed in adult rat brain following middle cerebral artery occlusion [108]. Up-regulation of Kv1.4, Kv2.1 and Kv4.2 expression both mRNA and protein levels is observed in amyloid peptide (25-35) -induced neurotoxicity [103]. In a Parkinson’s disease model using non-dopaminergic cortical neurons and midbrain dopamine neurons, it is shown that activation of oxidant-associated Kv-channel-dependent cell death pathway occurred upon 6-hydroxydopamine (a dopamine transporter inhibitor) application, which can be effectively inhibited by Kv channel blockers [109].

## KIR CHANNELS

**Fig. (3):**
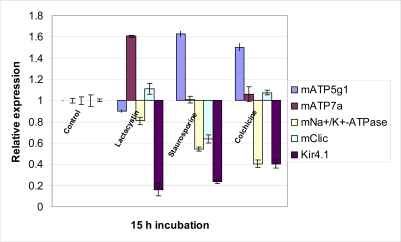
Analysis of the gene expression of potential ion channels at 15 h time of apoptotic onset in 1 µM lactacystin-, staurosporine- and colchicines-treated cultured murine cortical neurons.

Potassium channels dysfunction has been involved in the pathogenesis of AD. A recent study of AD model showed a specific augmentation in the delayed rectifier potassium current but not the transient outward current before the onset of beta-amyloid peptide(1-40)-induced neuronal apoptosis. Furthermore application of TEA, a Kv channel blocker or elevated extracellular potassium concentration, was able to attenuate this cell death and inhibit the appearance of apoptotic morphological events, such as chromatin condensation and cytochrome c release with caspase activation, suggesting early phase activation of potassium channels may be critical in the subsequent propagation of the apoptotic signaling pathway [148]. Further compelling evidence from the neuroprotective effect of TEA and high extracellular K^+^ concentrations in staurosporine- , ceramide- and serum deprivation-mediated apoptosis through suppression of enhanced K^+^ current in cultured mouse cortical neurons enhance the importance of K^+^ efflux in apoptotic activation [137, 152, 153]. Furthermore, in cerebral granule neurons, it was shown that apoptosis could be induced by low but physiological extracellular K^+^ concentrations suggesting high extracellular K^+^ levels are required for normal development and survival of neurons [29]. However, contrasting evidence are demonstrated in malignant astrocytoma cell lines U87 and A172, where 4-AP, a Kv channel blocker, induced apoptosis and inhibited the 4-AP-sensitive outward rectifier K^+^ current, effects which are opposite that of TEA [23]. Since Kir channel activity is positively regulated by extracellular K^+^ concentrations and Em hyperpolarization [90], involvement of Kir channels in the inhibition of apoptosis is justified. Our real-time PCR data (Fig. **3**) indicated a downregulation of Kir4.1 channel gene expression occurring at 15h onset of apoptosis induced by lactacystin, staurosporine and colchicine respectively, thereby suggesting that inactivation of Kir channels activity is required for the outward delayed rectifier potassium current to take place, resulting in K^+^ efflux since the counteracting inward movement of K^+^ is inhibited.

## K2P CHANNELS

K2P channels, a type of potassium leak/background channels, are independent of regulation by intracellular messengers such as arachidonic acid or ATP, transmembrane voltage fluctuation and cytoskeleton structural changes and open at rest. They have been found to be abundantly expressed in the brain. Unlike other K^+^ channels, K2P channels comprise of two P domains in tandem. K2P channels, compring of TALK, TWIK and TASK are recently implicated in the early enhancement of K^+^ outward current during apoptosis in cultured rat cerebellar granule neurons induced by low extracellular K^+^. Particularly TASK-3 ((TWIK)-related acid-sensitive-3) was shown to induce apoptosis after genetic transfer of TASK subunits into hippocampal neurons [104].

## K^+^-PERMEABLE IONOTROPIC RECEPTORS

Cytoplasmic K^+^ efflux can occur *via* pore-containing receptors other than K^+^ channels. It has been well established that N-methyl-D-aspartate (NMDA) receptors-mediated neuronal cell death is associated with Ca^2+^ and Na^+^ influx during excitotoxicity, eventually resulting in necrosis [24]. However, in a model of brain ischemia, cortical neurons exposed to NMDA lost substantial intracellular K^+^ and underwent apoptosis in medium containing reduced Na^+^ and Ca^2+^ (observed in ischemic brain tissue) [154]. This is possible as NMDA receptor-gated channels have been known to be permeable to ions such as K^+^, Na^+^ and Ca^2+^. The NMDA-induced outward K^+^ current could be inhibited with high extracellular K^+^ level, even in the absence of functional voltage-gated Ca^2+^ channels. This proved effective in reversing the apoptotic process. Loss of cellular K^+^ may present a prerequisite step in the activation of caspases and the central apoptotic cascade [6, 11, 26, 49, 83, 106, 131, 151, 153], explaining the apoptotic neuronal cell death observed after ischemia, and forming the ionic basis of NMDA-receptor-mediated apoptosis, which is apparently different from that of NMDA-receptor-mediated necrosis (comprises of Na^+^ and Ca^2+^ influx). Our microarray analysis further substantiated this hypothesis by showing a gene upregulation of ionotropic glutamate receptor, NMDA receptor 1 at 4.5 h and 7.5 h lactacystin treatment on cultured cortical neurons, precedent to apoptosis onset at 15 h (Table **1**). In contrast, a gene downregulation of ionotropic glutamate receptor, AMPA receptor 2 was observed, highlighting the important role played by NMDA receptors in mediating AVD.

## REGULATION OF POTASSIUM CHANNELS

Study conducted by [102] demonstrated that target soluble N-ethylmaleimide-sensitive factor attachment protein receptor (t-SNARE) proteins are involved in the pro-apoptotic promotion of K^+^ channel translocation to the cell surface, highly probable in the cell membrane fusion step. Intriguingly, t-SNARE proteins have also been implicated in the trafficking and/or surface expression of other plasma membrane channels; presumably ligand-gated ion channels [68, 75, 113, 135]. Dominant negative constructs of Kv2.1 not only prevent the K^+^ current efflux, but prove to be also neuroprotective [101]. Activity of K^+^ channels can be modulated by their tyrosine phosphorylation status, allowing direct control over the apoptotic process. In cortical neurons with tyrosine kinase-inhibition, Fas- and ceramide-induced apoptosis are able to be attenuated with suppression of N-type and delayed rectifier channel upregulation [152]. This is further substantiated by the discovery of essentiality of p38 activation for early K^+^ efflux in thiol-mediated neuronal apoptosis, and that inhibition of p38 kinase activity could inhibit this TEA-sensitive K^+^ current [84]. In contrast, in SH-SY5Y subjected to hypoxia and glucose deprivation, attenuation of cell death was achieved by tyrosine phosphorylation of Kv1.2 by VEGF [108]. As such, tyrosine phosphorylation may provide an important way in regulation of cell survival and activity of K^+^ channels.

Interesting, membrane rafts have been proposed to be involved in the regulation of ion channels during apoptosis, by formation of large ceramide-enriched platforms from small sphingolipid- and cholesterol-enriched membrane domains (Reviewed by [121]). These platforms are suggested to promote receptor clustering and rearrangement of cellular signaling proteins, therefore in the process bringing the ion channels to close proximity with their activators or separating them into individual functional channels.

## ROLE OF Na^+^/K^+^ ATPase

During basal state, a cell typically possesses a high intracellular K^+^ concentration (approximately 28-30 times higher than extracellular) and a low intracellular Na^+^ concentration. The continuous maintenance of the resultant ion gradients are essential for osmotic balance, cell volume regulation, Na^+^-coupled transport of nutrients and amino acids into cells, and restoration of resting membrane potential in excitable cell. This differential ion distribution is resulted from and maintained by the constitutive action of Na^+^/K^+^-ATPase. Na^+^/K^+^-ATPase, is a ubiquitous and indispensable transmembrane enzyme which poses as an electrogenic ion transporter in the plasma membrane of mammalian cells. The energy-dependent pump exchanges 3 Na^+^ ions into the extracellular matrix for every 2 K^+^ ions channeled into the cells, in the process utilizing 1 ATP molecule. This mode of movement results in a net efflux of ions, allowing the cells to compensate the colloidal osmotic pressure which arises as a result of an accompanied influx of water due to a passive inward movement of ions (K^+^, Na^+^). If uncompensated, this uncontrolled surge of water into the cells will eventually cause cell to swell and lyse. In addition to being ATP-dependent, Na^+^/K^+^-ATPase activity can be regulated by phosphorylation status, regulatory agents (endogenous ouabain-like substances [15, 30, 34] neurotransmitters such as dopamine (inhibitory) and norepinephrine (stimulatory) [42; 159] and hormones such as insulin [48], oxidant stress such as reactive oxygen species (ROS), [67] and by ionic distributions across the membrane [36, 67, 112]. Dysfunction of this pump imposes disastrous consequences on the cells, causing an instantaneous loss of intracellular K^+^ and accumulation of intracellular Na^+^, leading to membrane depolarization and a rise in intracellular free Ca^2+^ level, contributed by the opening of the opening of voltage-gated Ca^2+^-channels and reversed operation of the Na^+^-Ca^2+^ exchanger.

The reduction in functional or number of Na^+^/K^+^-ATPase has been observed in various chronic neurodegenerative disorders. For instance in AD brain, the Na^+^/K^+^-ATPase a3 subunit mRNA was approximately 30–45% lower than in that of controls [20]. Similarly, significant downregulation of Na^+^/K^+^-ATPase mRNA was observed from our real-time PCR data at the onset of lactacystine-, staurosporine- and colchicine-mediated apoptosis (Fig. **3**). Since compelling evidence demonstrated that Na^+^/K^+^-ATPase is involved in the rectification of perturbed K^+^ and Na^+^ ion homeostasis during apoptosis, a loss of Na^+^/K^+^-ATPase function is naturally required for programmed cell death to proceed. A recent study indicated that lack of tyrosine phosphorylation can inhibit the activity of Na^+^/K^+^-ATPase in cortical neurons, eventually resulting apoptosis [133]. 

As previously discussed, the pro-apoptotic effect of K^+^ efflux can be mediated by the voltage-gated K^+^ channels and the ionotropic glutamate receptors [89, 140, 151-154]. In the case of Na^+^/K^+^-ATPase dysfunction, K^+^ efflux would be further enhanced, causing a even more substantial loss of intracellular K^+^, approximately 50-80% depletion in ouabain-treated cells [96, 141]. As such, it can be inferred that K^+^ efflux occurred during AVD can be compensated as long as function of Na^+^/K^+^-ATPase is not compromised [149]. Therefore, normal intrinsic activity of Na^+^/K^+^-ATPase may provide a critical mean to evade cells from apoptosis by maintaining K^+^ homeostasis.

Ouabain, a cardiac glycoside, is commonly used to treat cells to induce inhibition of Na^+^/K^+^-ATPase. The molecular basis of ouabain cytotoxicity is due to Ca^2+^ influx and K^+^ efflux. Numerous studies using cultured cortical neurons demonstrated that activity of Na^+^/K^+^-ATPase was severely blocked at high ouabain concentrations resulting in momentary cell swelling followed by persistent progressive cell shrinkage together with loss of intracellular K^+^ ions [63, 119, 141]. The nature of cell death induced by Na^+^/K^+^-ATPase blockade showed a concurrent occurrence of apoptosis and necrosis. It is suggested that Na^+^/K^+^-ATPase dysfunction induced apoptosis by depleting cellular K^+^ and, simultaneously, caused necrotic injury within the same cells by increasing intracellular Ca^2+^ and Na^+^. Experimental evidence demonstrated that though blockade of individual K^+^, Na^+^ and Ca^2+^ channels could attenuate cell death, but a co-application of these blockers would confer additional neuroprotective effect [141]. On the contrary, a recent study by [52], showed that ouabain exhibited neuroprotection to rat cerebellar granule cells when exposed to low extracellular K^+^, which can trigger apoptosis.

Further evidence employing also cultured cortical neurons have shown that dysfunction of the Na^+^/K^+^ ATPase can result from an increased generation of ROS during cellular exposure to ceramide, serum deprivation and staurosporine [11].

## ROLE OF MITOCHONDRIAL K^+^-PERMEABLE CHANNELS

Mitochondria, is the ‘powerhouse’ in cells by playing central roles in meeting cellular energy demands through ATP production and in regulating calcium homeostasis. On the other hand, mitochondria are also involved in the trigger and propagation of cell death, prominently in apoptosis. This critical balance of life and death is maintained by subcellular compartmentalization. Mitochondrial dysfunction has been implicated in the pathogenesis of numerous neurodegenerative disorders such as AD, PD, Huntington’s disease and amyotrophic lateral sclerosis. This loss of normal mitochondrial activity can lead to degeneration of synapses, and eventually cell death. Studies have demonstrated that apoptotic cascades involving mitochondria are triggered in synaptic terminals, and in dendrites and axons, by several different stimuli comprising of the neurotransmitter glutamate, amyloid β-peptide, oxidative stress, and the bacterial alkaloid staurosporine [81,82]. Typical mitochondrial responses to the death stimuli include membrane depolarization, calcium uptake, oxyradical production, and release of cytochrome c with subsequent apoptosome formation resulting in caspase activation [81].

Recent studies have implicated the potential roles of mitochondrial ionic channels in apoptotic progression. Existence of MitoK channels has been confirmed in the mitochondrial inner membrane in neurons [27]. Though mitochondria are known to contain ATP-sensitive K^+^ (Mito-KATP) channels, their unavailability to electrophysiological analyses has been an obstacle to the through understanding of its true potential value in the participation of cell death progression [50]. Mitochondrial ATP-sensitive potassium (MitoKATP) channels are known to be involved in the modulation of inner membrane potential and oxyradical production, and that varying mitochondrial potassium fluxes can influence cytochrome c release. Evidence has demonstrated that activation of Mito-KATP can protect cultured neurons against hypoxic injury by a mechanism involving suppression of oxyradical production and prevention of cytochrome c release from mitochondria [74]. Neuroprotective effect of Mito-KATP has also been evident in dopaminergic neurons against toxin-induced death in a model relevant to PD [123]. Mechanistic events of how Mito-KATP exerts its protective effect has been unclear, but a recent study showed that activation of Mito-KATP by diazoxide increases the association of Bcl-2 with mitochondria, while prevents Bax translocation to mitochondria [74].

## ROLE OF CHLORIDE IONS

Maeno *et al.*[78] demonstrated that induction of AVD under normotonic conditions was coupled to facilitation of RVD, known to be attained by parallel operation of Cl^-^ and K^+^ channels, under hypotonic conditions. K^+^ outward release during AVD, generates a positive potential on the exterior, leading to concurrent Cl^−^ efflux down the electrochemical gradient to maintain homeostatic ionic balance and electroneutrality of the cell [8, 70, 78, 150]. This anion extrusion is further enhanced by the opening of the membrane Cl^-^ channels induced by the K^+^ efflux-induced hyperpolarization [156]. Cl^- ^is the most abundant anion within the cell, and its efflux is both favoured by the electrical and concentration gradients upon K^+^ release. In an effort to resolve the osmotic perturbation, cells extrudes water through aquaporins (water channels), resulting in cell shrinkage.

Trigger and/or augmentation of outwardly rectifying Cl^−^ currents (ORCC) is observed upon apoptotic stimulation in various cell type from Jurkat T cells [122], to non-pigmented ciliary epithelial cells [25]. Since Cl^-^ channels involvement in apoptosis have been highlighted in many cell types treated with apoptotic stimuli, an apoptotic role for Cl^-^ is highly feasible. Rapid outward Cl^-^ conductance can be triggered by apoptotic stimuli from either intrinsically by mitochondria or extrinsically by death receptor. Consequently, a protective effect against apoptosis is achieved in some peripheral cells using Cl^-^ channel blockers [95, 122].

ORCC that are involved in induction of AVD are demonstrated to be volume-sensitive, in which activation occurs upon cell swelling under non-apoptotic conditions. These anion channels are crucial in regulating the volume sensitivity, outward rectification, intermediate unitary conductance, low-field anion selectivity, broad-spectrum sensitivity to anion channel blockers and cytosolic ATP dependence [97]. [78] demonstrated that inhibition of volume-sensitive ORCC in neuronal, PC12, lymphoid and HeLa cells exposed to various apoptotic insults was able to inhibit AVD. A recent paper demonstrated that these volume-sensitive ORCC is involved in excitotoxic responses in mouse cortical neurons upon NMDA exposure [51]. However, much controversy exists over the identity of the volume-sensitive ORCC. CLC-3 is proposed to be the latest candidate protein involved in the trigger of volume-sensitive-like ORCC current induced by apoptotic stimuli [32]. The CLC channel family, a group of voltage-dependent Cl^−^ channels, is widely expressed in different types of cells. These channels are involved in the regulation of Cl^−^ homeostasis and cell volume under physiological and pathological conditions [28, 58, 118]. CLC-2 (inward rectifying) and CLC-3 (outward rectifying) channels are prominent members of CLC family that are found in cortical neurons and presumably to reside in the plasma membrane although there is much debate over their exact cellular distribution [14, 32, 45, 99, 139]. Activation of CLC-2 and CLC-3 channels are detected in cultured cortical neurons upon induction of AVD and apoptosis by staurosporine, C2-ceramide and serum deprivation respectively [136]. Microarray analysis demonstrated an initial close to 2-fold rise in CLC-3 mRNA levels during 4.5 h and 7.5 h lactacystin-treated neurons, prior to onset of apoptosis, which subsequently decreases drastically at 24 h and 48 h treatment timepoints (Table **1**).

Real-time PCR data showed a variable gene expression of mClic, a member of the CLIC intracellular chloride channel which showed localization to the cytoplasmic and inner mitochondrial membrane on different models of neuronal death (Fig. **3**). mClic demonstrated a significant mRNA downregulation in staurosporine-mediated apoptosis in cultured cortical neurons. On the contrary, an insignificant mClic gene upregulation was observed in lactacystin- and colchicine-mediated neuronal apoptosis. Upregulation of mClic mRNA expression is further substantiated by the microarray analysis demonstrating temporal increase of mClic1 over a 48 h time course in lactacystin-mediated neuronal death (Table **1**).

However, recently surfacing evidence denies CLC channels to be the volume-sensitive ORCC [132]. CLC-3 knockout mice have demonstrated functional expression of volume-sensitive ORCC independent of CLC-3 expression [132]. Furthermore, staurosporine-induced caspase-3 activation, DNA laddering and apoptosis occurred in both CLC-3 null mutant and wild type mouse cardiomyocytes, though decrease in cell survival was slight but significant in CLC-3 deficient phenotype. Nevertheless, similar extent of pharmacological apoptotic inhibition was observed in both phenotypes [134], a clear indication that CLC-3 are morphologically distinct from the protein constituting the pore of volume-sensitive ORCC, and its identity remains to be elucidated. CLC-3 channels prove to possess some role in apoptosis though.

Effectiveness of Cl^-^ channel blockers against apoptosis are inconsistent and controversial in numerous studies. Study conducted by [115] using primary rat cortical neuronal cultures demonstrated that application of Cl^-^ channel blocker was more effective than that of K^+^ and Na^+^ channel blockers against staurosporine-induced apoptosis, and this inhibition of apoptosis followed a dose-dependent manner, suggesting that Cl^-^ rather than K^+^ or Na^+^ plays a pivotal role in apoptosis. Recent findings conducted by [98] supported the evidence from previous study and observed that inhibition of volume-sensitive Cl^-^ channels could prevent the occurrence of AVD and downstream apoptotic events in ischemia-reperfusion-induced apoptosis in cardiomyocytes and brain neurons. In contrast, data from [136] proved otherwise and demonstrated that Cl^-^ channel blockers were able to inhibit AVD in cortical neurons treated with staurosporine, however typical apoptotic morphological changes such as chromatin condensation and DNA fragmentation were still exhibited resulting in programmed cell death. Only application of K^+^ channel blockers, TEA and clofilium was able to successfully suppress AVD occurrence together with other features of apoptosis [136]. This implies that the Cl^-^ channels are mainly involved in cell volume regulation in the event of apoptosis by triggering cell shrinkage, in contrast to the K^+^ channels which plays a decisive role in central apoptotic events such as caspase activation and DNA fragmentation. Moreover, the cell shrinkage is not a crucial step in the activation of the apoptotic cascade such that apoptosis can still proceed in the absence of cell shrinkage. 

## ROLE OF SODIUM IONS

Na^+^ ion is given very little attention by researchers in understanding it functional role in apoptosis since it is present intracellularly at low concentrations. However, consistently observed in various apoptotic stimuli-induced cell death models, an early transient augmentation of intracellular Na^+^ associated with plasma membrane depolarization preceded cell shrinkage and other features of apoptosis [10, 33, 61, 114]. It is proposed in rat hepatocytes, that the initial rise in intracellular Na^+^ during apoptosis was due to a shrinkage-activated sodium conductance in accordance to the RVI response [7]. This elevation of intracellular Na^+^ is short-lived and subsequently followed by a drop in both intracellular K^+^ and Na^+^ levels. The initial rise in intracellular Na^+^ is suggested to be contributed by two major sources: the inhibition of the Na^+^/K^+^-ATPase and the opening of the plasma membrane Na^+^ channels. Neuronal apoptosis has been shown to result from voltage-gated Na^+^ channel activation leading to Na^+^ entry into the cells [5]. This is further supported by the induction of apoptosis in hippocampal neurons by veratri-dine, a Na^+^ channel opener, due to the constitutive activation of voltage-gated Na^+^ channels leading to a prolonged state of depolarization [62]. Co-treatment of veratridine-treated neurons with AM-36, a Na^+^ channel inhibiting free radical scavenging drug, was able to attenuate apoptosis by inhibiting rise in intracellular Na^+^ and reactive oxygen species production [19]. On the other hand, application of a Na^+^ channel blocker, tetradotoxin, was able to block hypoxia-induced neuronal apoptosis [62]. 

## CONCLUSION

Apoptosis is the predominant form of cell death pathway that most cells adopted in numerous neurodegenerative disorders. Apoptosis is a highly sophisticated and elaborate mode of cell death, which requires precise and coordinated cooperative among different cellular signaling pathways, to ensure the continuation of the transmission of death signal. It is an elegant and isolated mechanism of induction of cell death without causing massive killing of neighboring cells. Apoptosis is a highly regulated process with numerous feedback loops be it positive or negative at various stages, ensuring that alternative signaling cascades are present to compensate for the deficiency or inefficiency of any one signaling pathways. As such, numerous classical morphological features of cells undergoing apoptosis, e.g. cell shrinkage, membrane blebbing, chromatin condensation and DNA fragmentation, though are frequently observed, their occurrences are not universal in all apoptotic cells. 

Though it has been shown that apoptosis can still occur in cells in the absence of AVD, the contribution of the underlying movements of ions and water during AVD to ensure the smooth proceeding of apoptosis cannot be denied. In this review article, we have looked into the rationale and importance of the individual ion and water movements in the event of AVD, an event precedent to apoptosis, particularly with respect to cells of CNS. Although controversy exists with respect to the mitochondrial AQPs expression and their involvement in apoptosis, the importance of plasma membrane AQPs in apoptosis causes no doubt. The need for functional AQPs during AVD and for the eventual progress of apoptosis is clearly evident. By understanding the significance of the ion and water fluxes during pathological states, in correlation to that during physiological state, presents an opportunity for identification of therapeutic targets directed at the initial stage of apoptosis, i.e. AVD to allow manipulation of apoptosis or apoptosis-related diseases, thus allowing a more effective inhibition of neuronal death.

## Figures and Tables

**Table 1:** Differentially Expressed Genes from Microarray Analysis of Murine Primary Cortical Neurons Treated with 1 µM Lactacystin

Genbank No.	Gene Title	Gene Symbol	4.5h	7.5h	24h	48h
**Water channels**						
U88623	Aquaporin 4	Aqp4	-1.59 ± 0.08	-6.24 ± 0.09	-6.17 ± 0.1	1.22 ±0.16
Na+/K+ ATPase						
AW123952	ATPase, Na+/K+ transporting, alpha 1 polypeptide	Atp1a1	-1.26 ± 0.1	2.16 ± 0.09	1.13 ± 0.18	1.46 ± 0.14
AI829697	ATPase, Na+/K+ transporting, alpha 2 polypeptide	Atp1a2	1.02 ± 0.08	-1.64 ± 0.07	-3.57 ± 0.09	-1.03 ± 0.26
**Chloride channels**						
AF029347	Chloride channel 3	Clcn3	2.23 ± 0.07	1.76 ± 0.07	1.14 ± 0.1	-1.26 ± 0.16
AF109905	Chloride intracellular channel 1	Clic1	1.45 ± 0.11	1.76 ± 0.09	3.64 ± 0.18	3.2 ± 0.45
**Ionotropic receptors**						
L32372	Glutamate receptor, ionotropic, AMPA2 (alpha2)	Gria2	2.13 ± 0.07	1.18 ± 0.09	-1.15 ± 0.11	-1.18 ± 0.11
D10028	Glutamate receptor, ionotropic, NMDA1 (zeta1)	Grin1	-1.02 ± 0.13	2.3 ± 0.1	-1.68 ± 0.16	-1.73 ± 0.17
**Potassium channels**						
AI850484	Potassium voltage-gated channel, shaker-related subfamily, member 3	Kcna3	2.06 ± 0.16	1.22 ± 0.12	-1.16 ± 0.1	-1.14 ± 0.12
AB000503	Potassium voltage-gated channel, subfamily Q, member 2	Kcnq2	-2.33 ± 0.08	-1.9 ± 0.07	1.03 ± 0.15	-1.24 ± 0.1
**Solute carrier families**						
D43797	Solute carrier family 1 (neuronal/epithelial high affinity glutamate transporter, system Xag), member 1	Slc1a1	1.15 ± 0.25	1.88 ± 0.17	-2.14 ± 0.15	-2.42 ± 0.15
U75215	Solute carrier family 1 (glutamate/neutral amino acid transporter), member 4	Slc1a4	1.4 ± 0.06	2.41 ± 0.1	-1.03 ± 0.26	-1.52 ± 0.1
AA062013	Solute carrier family 25 (mitochondrial carrier; adenine nucleotide translocator), member 5	Slc25a5	-1.64 ± 0.08	-2.59 ± 0.06	-1.06 ± 0.17	1.06 ± 0.13
U76009	Solute carrier family 30 (zinc transporter), member 3	Clc30a3	-2.52 ± 0.08	-2.06 ± 0.07	-1.26 ± 0.11	-1.2 ± 0.1
AV278013	Solute carrier family 4, sodium bicarbonate cotransporter, member 7	Slc4a7	-3.75 ± 0.07	-2.01 ± 0.07	-1.12 ± 0.1	1.02 ± 0.16
AF020195	Solute carrier family 4 (anion exchanger), member 4	Slc4a4	-1.3 ± 0.13	-3.96 ± 0.11	-1.48 ± 0.13	1.46 ± 0.15
AV230927	Solute carrier family 6 (neurotransmitter transporter, serotonin), member 4	Slc6a4	-1.56 ± 0.12	-2.23 ± 0.08	-1.93 ± 0.13	1.25 ± 0.13
U25739	Solute carrier family 23 (nucleobase transporters), member 3	Slc23a3	-2.48 ± 0.12	-2.94 ± 0.08	-1.25 ± 0.1	-1.36 ± 0.1
AW048729	Solute carrier family 5 (sodium-dependent vitamin transporter), member 6	Slc5a6	-2.48 ± 0.07	-2.76 ± 0.08	-1.3 ± 0.13	-1.24 ± 0.12
